# Factors Affecting the Career Continuation of Newly Graduated and Reinstated Dental Hygienists Who Participated in a Technical Training Program in Japan

**DOI:** 10.3390/ijerph192013360

**Published:** 2022-10-16

**Authors:** Kanako Noritake, Katsuo Oshima, Hideki Fukuda, Rumi Tano, Akiko Oshiro, Hiroshi Nitta, Hiroko Miura

**Affiliations:** 1 Oral Diagnosis and General Dentistry, Tokyo Medical and Dental University Hospital, Bunkyo-ku, Tokyo 113-8510, Japan; 2 Department of Dental Technology, The Nippon Dental University College at Tokyo, Chiyoda-ku, Tokyo 102-8159, Japan; 3 National Institute of Public Health, Wako 351-0197, Japan; 4 Department of Oral Health Promotion, Graduate School of Medical and Dental Sciences, Tokyo Medical and Dental University, Tokyo 113-8510, Japan; 5 General Dentistry, Graduate School of Medical and Dental Sciences, Tokyo Medical and Dental University, Bunkyo-ku, Tokyo 113-8510, Japan; 6 Division of Disease Control and Epidemiology, School of Dentistry, Health Sciences University of Hokkaido, Ishikari-gun 061-0293, Japan

**Keywords:** dental hygienists, continuous employment, career outlook, career break, job satisfaction

## Abstract

This study aimed to identify (1) what newly graduated dental hygienists and reinstated dental hygienists consider important for preventing early turnover in their own professions and for encouraging them to continue in the profession more generally and (2) relevant factors among hygienists intending to continue working in the field. An anonymous, self-administered questionnaire survey was distributed to 215 Japanese dental hygienists who participated in the technical training programs (response rate: 72.6%). Of them, 143 participants were classified into two groups: newly graduated (NGDH, *n* = 32) and those reinstated to work (RDH, *n* = 111). Follow-up for lack of skills was most often selected as important for preventing early turnover among both groups and follow-up for lack of knowledge was significantly selected in the RDH group (*p* < 0.001). Regarding factors important for career continuation, NGDHs significantly selected gaining job satisfaction, whereas RDHs significantly selected working support. Relevant factors contributing to participants’ intentions to continue in the profession were job satisfaction (NGDH, OR = 8.37; RDH, OR = 8.83), career outlook (RDH, OR = 3.11), and job turnover experience because of marriage and parenting (RDH, OR = 2.70), thereby suggesting the importance of raising awareness regarding career progression and job-related rewards among dental hygienists, their educators, and the government through ongoing career education.

## 1. Introduction

In recent years, increasing evidence has confirmed that the perioperative management of oral function reduces the incidence of postoperative complications [1,2], as well as the relationship between oral health/frailty and systemic health/disease, such as cardiovascular disease, diabetes, rheumatoid arthritis, and sarcopenic dysphagia, among others [3,4,5,6,7,8]. Dental hygienists support the promotion of dental and oral health across generations and are responsible for improving oral function and hygiene; thus, they are expected to play an increasingly important role, requiring specific skill sets, such as approaches to support older institutionalized residents and home care providers, which cannot be provided by conventional dental clinics and dental hospitals [9,10,11]. In recent years, the receipt of regular dental checkups has been strongly related to the number of dental hygienists in a dental clinic and the length of time spent on dental health guidance [12].

Internationally, women dominate the dental hygiene profession; this trend remains true in Japan, where 99.95% of all dental hygienists are women [13]. Notably, Japan has the most dental hygienists worldwide [14]; however, as of 2018, the employment rate of licensed dental hygienists has become relatively low (46.0%) [13,14]. This indicates that many registered dental hygienists do not work in these roles, creating a shortage of dental hygienists in Japan [14,15,16]. As for the situation in other countries regarding the labor shortage of dental hygienists, in the U.S., supply exceeded demand for the country as a whole, but remained insufficient in some states [17]. However, the latest reports indicated that COVID-19 has caused an estimated 8% reduction in dental hygienist employment in the U.S. [18]. The supply of dental hygienists in Canada has also been increasing; however, there was a nearly twofold difference in supply rates across regions [19]. Therefore, the shortage of dental hygienists is not a problem limited to Japan but could serve as a precedent for other countries. Furthermore, nurses often experience issues with labor shortages, which is yet again a global problem and not one limited to Japan [20,21]. Given this background, expectedly, various studies have reported on nurses’ intentions to leave the profession [21,22,23].

Career breaks are common for dental hygienists worldwide, primarily because of childbirth and parenting [24,25]. In addition, professional reality shock, feelings of anxiety and loss, and the desire to give up in response to a gap between one’s expectations and experience concerning work content, human relations, and other work-related factors can lead to early retirement and turnover, especially in the period immediately after obtaining a license [26]. Reality shock has been reported to occur among new dental hygienists in Japan during their first year of employment [27] as well as in Japanese nurses [28,29], making early retirement a serious concern [14]. The number of people who leave the dental hygiene profession within three to four years of graduating from a training institution and begin working as a hygienist has increased in their 50s [30]. Moreover, the labor force of dental hygienists tends to rapidly decline as hygienists reach their late 20s and early 30s, and continues to decline further thereafter, indicating barriers to dental hygienists returning to work after a significant life event (e.g., childbirth) [14,31].

Therefore, in Japan, measures are focused on preventing dental hygienists from leaving work immediately after obtaining a license and supporting their return to work after a leave of absence [32]. In 2017, the Ministry of Health, Labor, and Welfare (MHLW) established dental hygienist training centers to provide technical training [14]. Three dental hygienist training institutions were established in the 2019 fiscal year. The purpose of these centers is to support the return to work of dental hygienists who have left the profession for parenting, nursing care, or other reasons, and promote the acquisition of basic clinical skills for newly licensed dental hygienists, thus decreasing the attrition in the profession [14].

Despite attempts to support these two groups of hygienists, dental hygienists in leadership positions believe that the nature of the support needed for these two groups differs [32]. To date, there has been no discussion from the perspective of the parties involved regarding such differences—specifically, what dental hygienists in these two groups believe is necessary to continue working, as well as what factors affect dental hygienists who intend to continue working without a career break.

To address this gap, we conducted a survey of participants in a training program that was provided by the training centers set up by the MHLW, dividing them into newly graduated and reinstated dental hygienists. Our study was conducted with two objectives: (1) to identify what participants consider important for dental hygienists to continue in the profession and (2) to identify relevant factors among survey participants who intended to continue in the profession. The results contribute to the MHLW’s planning of policies necessary for dental hygienists to continue in the profession.

## 2. Materials and Methods

### 2.1. Target

All dental hygienists who completed their training programs at any of the three dental hygienist centers, (A, B, and C) commissioned by the MHLW, between January 2018 and March 2020 were eligible for this survey. A total of 215 dental hygienists who had attended three dental hygienist training centers were targeted. (A: *n* = 110, B: *n* = 64, C: *n* = 41). Based on the MHLW’s survey of job turnover rates within three years of graduation [14], the definition of the newly graduated dental hygienists in this survey was set at less than three years after obtaining a license. Therefore, those who had been licensed for less than three years were classified into the “newly graduated dental hygienist” (NGDH) group, whereas those who had been approved for more than three years and had been out of work or returned to work for less than three years were classified into the “reinstated dental hygienist” (RDH) group.

### 2.2. Survey Items

An anonymous self-administered questionnaire survey was conducted by postal mail over three weeks from 2019 to 2020, with the cooperation of the staff of the three centers. The questionnaire was in Japanese, and questions and response options were designed based on previous studies’ survey content and questions regarding the employment of dental hygienists in Japan [13,33,34,35]. The questionnaire included questions regarding (1) participant demographics (e.g., age, years since qualification, current work status [full-time employment, part-time employment, job-seeking, or other], workplace [dental clinic, hospital, government, nursing home, home visitation, or other], and years of absence from the profession [RDH only]); (2) what is important to prevent early job turnover (select top three from six items); and (3) what is important to continue in the profession as a dental hygienist (select top three from nine items). In addition, participants were asked questions regarding (4) items related to dental hygienists (e.g., job satisfaction, intention to continue in the profession, and career outlook); and (5) reasons for leaving the profession in the past (marriage and parenting, job did not appeal to me, deterioration of workplace relationships, job description does not fit, or other) (RDH only). These sections targeted relevant factors among survey participants who intended to continue in the profession.

### 2.3. Analyses

For the items about what participants considered important for continuing in the profession as dental hygienists, the responses of the two groups to each question were compared using Pearson’s chi-square test.

To identify relevant factors among survey participants who intended to continue in the profession, multiple logistic regression analysis (forced entry method) was conducted. The dependent variable was the intention to continue in the profession in each group (“Willing to work as a dental hygienist for all my working life”; 0: no, undecided, 1: yes). The following were used as independent variables: rewarding (“Being a dental hygienist is rewarding”; 0: no, undecided, 1: yes), career outlook (“Having a career outlook as a dental hygienist”; 0: no, somewhat no, 1: yes, somewhat yes), parenting (“The reason for leaving job was marriage and parenting”; 0: no, 1: yes), and unappealing (“The reason for leaving job was because the job didn’t appeal to me”; 0: no, 1: yes). The latter two variables were only for the RDH group.

Statistical significance was set at *p* < 0.05. IBM SPSS Statistics for Windows (version 23.0; IBM Corp., Armonk, NY, USA) was used to perform all the statistical analyses.

### 2.4. Ethical Considerations

This research was conducted after receiving ethical approval from the National Institute of Public Health, Japan (approval number: NIPH-IBRA#12254).

## 3. Results

### 3.1. Characteristics of the Survey Participants

The collection rate for the postal survey was 72.6% (*n* = 156). Of the participants, 13 had been licensed for more than three years and had no history of job turnover; thus, they were not included in this analysis. Hence, 143 participants were included in the final analysis.

Of the 143 participants analyzed, 32 had been licensed for less than three years and were classified into the NGDH group, whereas 111 were licensed for more than three years and were currently or had been out of the workforce, and thus, were classified into the RDH group.

Table 1 provides demographic information regarding the participants. The mean age of all participants was 44.0 years. The mean age in the NGDH group was 31.7 years (range 22–56), whereas that of the RDH group was 47.6 years (range 27–69). The mean number of years since receiving hygiene licensure was 20.4 years overall, whereas the mean number of years was 1.4 years (range 0–3) and 25.7 years (range 5–43) for the NGDH and RDH groups, respectively. Analyses showed that 33% of participants indicated they were working full-time, 51% indicated they were working part-time, and 16% indicated that they were seeking employment/other. The average length of turnover in the RDH group was 12.9 years (range 0.5–37).

### 3.2. Important Factors for Preventing Early Job Turnover and Continuing in the Profession

**Figure 1:**
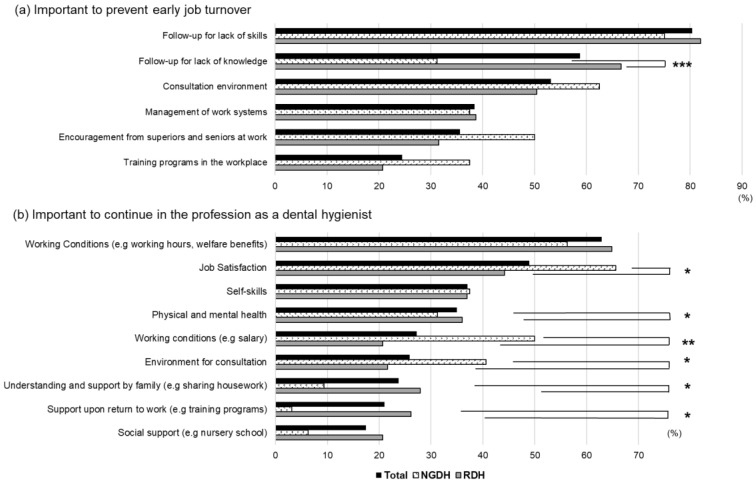
(**a**) Important items to prevent early job turnover, and (**b**) important items to continue in the profession as a dental hygienist. NGDH: new graduate dental hygienists, RDH: reinstated dental hygienists. * *p* < 0.05, ** *p* < 0.01, *** *p* < 0.001.

The participants selected three items that they thought were important for preventing early turnover in their own situation. Follow-up for lack of skills and knowledge, and consultation environment were selected most frequently (in that order); and follow-up for lack of knowledge was significantly selected in the RDH group (*p* < 0.001) (Figure 1a). They also selected items that they thought were important for continuing in the profession as a dental hygienist. Working conditions, job satisfaction, and self-skills were most frequently selected, in that order. Job satisfaction, salary, and consultation environment were significantly associated with the NGDH group (*p* < 0.05), whereas physical and mental health, family understanding, and support when returning to work were significantly associated with the RDH group (*p* < 0.05) (Figure 1b).

### 3.3. Analysis of Factors Relevant to the Intention to Continue in the Profession

Table 2 shows the results of the bivariate analysis of participants who intended to continue in the profession because they found it rewarding and those who intended to continue because of the career outlook, and their reasons for leaving. Significant associations were found between the groups (*p* < 0.05).

The results of the multiple logistic regression analysis showed that the proportion of those with the intention to continue in the profession in the NGDH group was significantly higher for those who found being a dental hygienist rewarding (odds ratio [OR] = 8.37, 95% confidence interval [CI] = 1.25–56.13, *p* = 0.029). In the RDH group, the proportion of participants with an intention to continue in the profession was significantly higher among those who found being a dental hygienist rewarding (OR = 8.83, 95% CI = 2.15–36.23, *p* = 0.003), had a career outlook (OR = 3.12, 95% CI = 1.19–8.14, *p* = 0.02), and had left the job previously for parenting (OR = 2.70, 95%CI = 1.01–7.20, *p* = 0.048) (Table 3).

## 4. Discussion

This is the first multicenter survey of dental hygienists who participated in a technical training program specifically designed for new graduates and returnees in Japan. The results allowed us to identify the support needs of each of the two target groups. As the most important factor in preventing early job turnover, follow-up training for lack of skills was selected for both targets. The next selected factor was follow-up for lack of knowledge, which was significantly higher in the RDH group than the NGDH group. Concerns regarding the lack of skills and knowledge among dental hygienists have been echoed in several previous studies. A nationwide survey of final-year dental hygiene students in Japan reported that the most common concern regarding employment after graduation was a lack of skills and knowledge, accounting for about half of the participants [36]. Anxiety about lack of skills and knowledge may not arise after becoming a dental hygienist, but rather before becoming a dental hygienist. Similarly, in surveys of dental hygienists in Japan, 30–47% of them also indicated a lack of their own skills as a barrier to returning to work [37,38]. Furthermore, these studies focused on Japan, and no reports from other countries were found in our literature review. Saito et al. reported that perceptions such as motivations for and expectations of being a dental hygienist were significantly lower among Japanese dental hygiene students than among Canadian students [39]; thus, this phenomenon may be unique to Japan.

Working conditions are most often selected by RDHs as a factor impacting their intention to continue working as a dental hygienist. This seems to be related to the difference in employment status between NGDHs and RDHs in this study; NGDHs were more likely to work full-time, whereas RDHs tended to work part-time. This fact is also consistent with the results of a previous study in Japan, which found that fewer than half of the participants aged 30 years or older wanted to work full-time [13]. This is also consistent with studies from other countries [24,25]. For instance, most participants who returned to work in the UK also returned to part-time employment [25], and dental hygienists with children in Australia reported working significantly shorter hours following their return to work [24]. Thus, job turnover because of life events such as marriage and parenting is a global issue. In the RDH group of this study, the mean number of years away from work was 12.9 years, which is longer than the average time away from work in the UK (30 months) [25] and Australia (20.1 months) [24]. This result reiterates that in many dental clinics in Japan, benefit systems have not been adequately established [40], suggesting that dental hygienists must resign rather than take a temporary leave of absence at the time of childbirth, causing a career break rather than an early return to work.

Several previous studies have reported that the work environment of Japanese dental hygienists must be improved [13,15,37,38]. According to the Global Gender Gap Report 2021, Japan’s overall score in 2021 on the Gender Gap Index was 120 out of 156 countries [40]. This gender gap makes it difficult for Japanese women to continue employment [13]. Therefore, returning to work within a relatively short period after leaving the dental hygiene profession—shortening the period of separation from the dental hygiene profession—is considered helpful in securing human resources and in developing the dental hygiene profession. Thus, we suggest that specific support for balancing childbirth and parenting with a career as a dental hygienist is necessary when returning to work. The multiple logistic analysis performed in this study also showed that the proportion of those in the RDH group with the intention to continue in the profession was significantly associated with those who left for marriage and parenting. This suggests that reasons for leaving the dental hygiene profession affect the intention to continue working when returning to the profession. In addition, some dental hygienists who are unavoidably leaving their jobs because of significant life events may actually want to continue working in their roles. Moreover, it is necessary to create a support system that allows these dental hygienists to return to work while maintaining a suitable work–life balance. Salary is also not overlooked in working conditions. NGDHs significantly selected salary as important to continue in the profession as a dental hygienist. According to a previous survey, salary was the fifth most selected multiple response reason for leaving the dental hygiene profession for dental hygienists not currently employed (the first being childbirth/parenting) and was the 13th leading reason [33], indicating that salary does not appear to be a direct reason for leaving. However, improving payment may also impact continued employment, as 72.5% of currently employed dental hygienists selected “improvement of labor conditions (base up, regular salary raise)” as the reason for what they would like to see improved at their current workplace [33].

Job satisfaction is related to the intention to continue working in both groups; in particular, it was most important to job continuity in the NGDH group and was significantly more important than in the RDH group. Previous studies in the U.S. have shown that the greater the job satisfaction, the less likely the choice to leave [41]. From the multiple logistic analysis of this study, the proportion of those in both groups who intended to continue in the profession was significantly associated with those who thought that being a dental hygienist was rewarding. These results suggest that another important need for the future is to create a system that allows dental hygienists to realize the satisfaction of their work before and immediately after graduation.

Career outlook was also identified as one of the factors relevant to RDHs’ intentions to continue working. In this study, a good career outlook was defined as having a future plan at work. Women with good career outlook are considered motivated to continue working in their organizations and are therefore considered capable of sufficient career development [42]. While there have been some studies on the careers of dental hygiene students [43] and employed dental hygienists [44], limited studies have investigated career outlook focusing on NGDHs and RDHs. The current results reiterate that having good career outlook is related to the intention to continue working, especially among those returning to work, suggesting that continuous career education may also help secure human resources in the field of dental hygiene.

One limitation of this study is that the training program has only recently been established, and the number of dental hygienists who have completed the training program is still relatively small. In particular, with approximately 7000 new dental hygiene graduates in Japan each year [43], we do not believe that the results of the NGDH group in this study are representative of the study population. It is assumed that the NGDH group in this study is relatively motivated to learn and is not representative of all new dental hygienists in Japan. Another limitation is that the comparison between the two groups is not age-corrected. Therefore, while the findings of this first survey of dental hygienists targeted for early turnover prevention support can be useful in identifying the necessary support, this group may not represent the general characteristics of new and returning dental hygienists in Japan.

## 5. Conclusions

This study found that NGDHs and RDHs are seeking the same types of support for preventing early job turnover, particularly, follow-up training for skills which they are lacking. However, what they considered necessary for continuity in the profession differed by life stage (e.g., job satisfaction and working support). We also found that the intention to continue working was related to having job satisfaction and career prospects as dental hygienists, highlighting the importance of raising awareness of potential career progression and incentives among dental hygienists themselves as well as their educators and the government through ongoing career education. These results suggest that the MHLW, in addition to directly providing technical training programs and continuing career education to the dental hygienists concerned, should establish an employment continuity support framework that is appropriate to life stages as a measure to ensure the professional continuity of dental hygienists.

## Figures and Tables

**Table 1:** Demographic information.

	Total	NGDH	RDH
	*n* = 143	*n* = 32	*n* = 111
		22.0%	78.0%
Mean age (SD)	44.0 (10.9)	31.7 (9.7)	47.6 (8.6)
Mean years since dental hygiene licensure (SD)	20.4 (13.0)	1.4 (0.8)	25.7 (9.3)
Work status			
Full-time employment (%)	47 (33%)	28 (88%)	19 (17%)
Part-time employment (%)	73 (51%)	4(12%)	69 (62%)
Job seeking/Other (%)	23 (16%)	0 (0%)	23 (21%)
Workplace (only those working responded)	***n* = 117**	***n* = 32**	***n* = 85**
Dental Clinic (including home visitation) (%)	93 (79%)	27 (84%)	66 (78%)
Hospital (%)	11 (9%)	5 (16%)	6 (7%)
Government/Nursing home/Others (%)	13 (11%)	0 (0%)	13 (15%)
Mean years of absence from work (SD) (only RDHs responded)			12.9 (8.0)

NGDH: new graduate dental hygienists, RDH: reinstated dental hygienists, SD: standard deviation.

**Table 2:** Bivariate analysis of those who found being a dental hygienist to be rewarding, and career outlook and reasons for leaving against the presence of continued intention.

	NGDH			RDH		
	Willing to Work as a Dental Hygienist for All My Working Life		*p*-Value	Willing to Work as a Dental Hygienist for All My Working Life		*p*-Value
Things related to dental hygienists	Yes	No/undecided		Yes	No/undecided	
Being a dental hygienist is rewarding	15 (47%)	7 (22%)	0.015	68 (61%)	27 (24%)	<0.001
Having a career outlook as a dental hygienist	12 (38%)	5 (16%)	0.039	37 (33%)	10 (9%)	0.005
Reasons for leaving job (only RDHs responded)				Yes	No	
For marriage and parenting	―	―	―	59 (53%)	24 (22%)	0.007
Because the job didn’t appeal to me	―	―	―	8 (7%)	12 (11%)	0.014

NGDH: new graduate dental hygienists, RDH: reinstated dental hygienists.

**Table 3:** Multiple logistic regression analysis showing the proportion of those with the intention to continue in the profession.

	NGDH			RDH		
Independent Variable	OR	95%CI	*p*-Value	OR	95%CI	*p*-Value
Being a dental hygienist is rewarding	8.37	1.25–56.13	0.029	8.83	2.15–36.23	0.003
Having a career outlook as a dental hygienist	4.67	0.88–24.69	0.069	3.12	1.19–8.14	0.02
Reason for leaving the job was marriage and parenting	-	-	-	2.70	1.01–7.20	0.048
Reason for leaving the job was because the job didn’t appeal to me	-	-	-	0.45	0.04–5.49	0.534

OR: odds ratio, CI: confidence interval, NGDH: new graduate dental hygienists, RDH: reinstated dental hygienists.

## Data Availability

Not applicable.

## References

[1] Iwata E., Hasegawa T., Yamada S.I., Kawashita Y., Yoshimatsu M., Mizutani T., Nakahara H., Mori K., Shibuya Y., Kurita H. (2019). Effects of perioperative oral care on prevention of postoperative pneumonia after lung resection: Multicenter retrospective study with propensity score matching analysis. Surgery.

[2] Pedersen P.U., Larsen P., Hakonsen S.J. (2016). The effectiveness of systematic perioperative oral hygiene in reduction of postoperative respiratory tract infections after elective thoracic surgery in adults: A systematic review. JBI Database Syst. Rev. Implement Rep..

[3] Shiraishi A., Yoshimura Y., Wakabayashi H., Tsuji Y. (2017). Poor oral status is associated with rehabilitation outcome in older people. Geriatr. Gerontol. Int..

[4] Meurman J.H., Bascones-Martinez A. (2021). Oral infections and systemic health—More than just links to cardiovascular diseases. Oral Health Prev. Dent..

[5] Kapila Y.L. (2021). Oral health’s inextricable connection to systemic health: Special populations bring to bear multimodal relationships and factors connecting periodontal disease to systemic diseases and conditions. Periodontol. 2000.

[6] Tavares M., Lindefjeld Calabi K.A., San Martin L. (2014). Systemic diseases and oral health. Dent. Clin. North Am..

[7] Cao W., Zhu A., Chu S., Zhou Q., Zhou Y., Qu X., Tang Q., Zhang Y. (2022). Correlation between nutrition, oral health, and different sarcopenia groups among elderly outpatients of community hospitals: A cross-sectional study of 1505 participants in China. BMC Geriatr..

[8] de Sire A., Ferrillo M., Lippi L., Agostini F., de Sire R., Ferrara P.E., Raguso G., Riso S., Roccuzzo A., Ronconi G. (2022). Sarcopenic dysphagia, malnutrition, and oral frailty in elderly: A comprehensive review. Nutrients.

[9] Nomura Y., Ohara Y., Yamamoto Y., Okada A., Hosoya N., Hanada N., Takei N. (2021). Dental hygienists’ practice in perioperative oral care management according to the Japanese dental hygienists survey 2019. Int. J. Environ. Res. Public Health.

[10] Wong F.M.F., Ng Y.T.Y., Leung W.K. (2019). Oral Health and Its Associated Factors Among Older Institutionalized Residents-A Systematic Review. Int. J. Environ. Res. Public Health.

[11] Salmi A., Komulainen K., Nihtila A., Tiihonen M., Nykanen I., Hartikainen S., Suominen A.L. (2022). Eating problems among old home care clients. Clin. Exp. Dent. Res..

[12] Inoue Y., Shimazaki Y., Oshiro A., Zaitsu T., Furuta M., Ando Y., Miyazaki H., Kambara M., Fukai K., Aida J. (2021). Multilevel analysis of the association of dental-hygienist-related factors on regular dental check-up behavior. Int. J. Environ. Res. Public Health.

[13] Miura H., Tano R., Oshima K., Usui Y. (2021). Analysis of factors related to working status of dental hygienists in Japan. Int. J. Environ. Res. Public Health.

[14] Japan Dental Hygienists Association Report on the Study Group on Securing Human Resources for Dental Hygienists and Support for Return to Work. https://www.jdha.or.jp/pdf/outline/fukusyokusien.pdf.

[15] Okada A., Nomura Y., Ohara Y., Yamamoto Y., Hosoya N., Hanada N., Takei N. (2021). Factors affecting the reinstatement of the Japanese dental hygienist: A Japanese dental hygienist survey conducted in 2019. Int. J. Environ. Res. Public Health.

[16] Murai A., Nishikiori R., Jin K. (2020). Investigation of supply and demand for dental hygienists in Japan. J. Osaka Odontol. Soc..

[17] U.S. Department of Health and Human Services, Health Resources and Services Administration, National Center for Health Workforce Analysis (2015). National and State-Level Projections of Dentists and Dental Hygienists in the U.S., 2012–2025.

[18] Gurenlian J.R., Morrissey R., Estrich C.G., Battrell A., Bessner S.K., Lynch A., Mikkelsen M., Araujo M.W.B., Vujicic M. (2021). Employment patterns of dental hygienists in the United States during the COVID-19 Pandemic. Am. Dent. Hyg. Assoc..

[19] Marchildon G.P., Allin S., Merkur S. (2020). Canada: Health System Review. Health Syst. Transit..

[20] Cheng L., Cui Y., Chen Q., Ye Y., Liu Y., Zhang F., Zeng W., Hu X. (2020). Paediatric nurses’ general self-efficacy, perceived organizational support and perceived professional benefits from Class A tertiary hospitals in Jilin province of China: The mediating effect of nursing practice environment. BMC Health Serv. Res..

[21] Yamaguchi Y., Inoue T., Harada H., Oike M. (2016). Job control, work-family balance and nurses’ intention to leave their profession and organization: A comparative cross-sectional survey. Int. J. Nurs. Stud..

[22] Nantsupawat A., Kunaviktikul W., Nantsupawat R., Wichaikhum O.A., Thienthong H., Poghosyan L. (2017). Effects of nurse work environment on job dissatisfaction, burnout, intention to leave. Int. Nurs. Rev..

[23] Heinen M.M., van Achterberg T., Schwendimann R., Zander B., Matthews A., Kozka M., Ensio A., Sjetne I.S., Moreno Casbas T., Ball J. (2013). Nurses’ intention to leave their profession: A cross sectional observational study in 10 European countries. Int. J. Nurs. Stud..

[24] Hopcraft M., McNally C., Ng C., Pek L., Pham T.A., Phoon W.L., Poursoltan P., Yu W. (2008). Working practices and job satisfaction of Victorian dental hygienists. Aust. Dent. J..

[25] Gibbons D.E., Corrigan M., Newton J.T. (2001). A national survey of dental hygienists: Working patterns and job satisfaction. Br. Dent. J..

[26] Kramer M. (1974). Reality Shock: Why Nurses Leave Nursing.

[27] Kimura N., Sumida Y., Hiyama K., Fukushima M. (2015). Reality shock and growth process of new dental hygienists in university dental hospital: Qualitative study on change of trouble and critical mind. J. Japan Soc. Dent. Hyg..

[28] Suzuki E., Itomine I., Kanoya Y., Katsuki T., Horii S., Sato C. (2006). Factors affecting rapid turnover of novice nurses in university hospitals. J. Occup. Health.

[29] Ikeda S., Matsueda M. (2020). A Survey of Relationships Between Job Stressors, Degree of Mental Health, Organizational Climate and the Identity of Newly Graduated Nurses. J. UOEH.

[30] Jin K., Nakatsuka M., Maesoma A., Wato M., Uene M., Doi T., Kataoka K., Miyake T., Komasa Y. (2017). Employment status of dental hygienists in Japan. J. Osaka Dent. Univ..

[31] Aida J., Kusama T., Igarashi A., Koseki T., Kosaka K., Hitomi S., Watanabe C. (2021). Factors related to turnover prevention and return to work for dental hygienists: Stress models and differences in perceptions compared with dentists. J. Dent. Health.

[32] Ohara Y., Uehara H., Shimatani K., Tamura K., Komori T., Okada M., Kobayashi M., Takei N., Tsuruta J. (2021). Challenges at work place among new graduate and reinstatement dental hygienists -text mining analysis of challenges from a teaching dental hygienist. J. Japan Soc. Dent. Hyg..

[33] Japan Dental Hygienists Association (2020). Reports of Actual Working Condition of Dental Hygienists.

[34] Wada K., Takei N., Kanazawa N., Miyoshi T., Noritake K. (2019). Working conditions of dental hygienists employed in Japanese hospitals. Int. J. Clin. Prev. Dent..

[35] Usui Y., Miura H. (2015). Workforce re-entry for Japanese unemployed dental hygienists. Int J Dent Hyg.

[36] Tano R., Miura H., Noritake K., Oshima K., Mizugai H., Fukuda H. (2021). The employment number of years in the first job desired and veiw of employment in the dental hygienist student of final year by the nationwide survey in Japan. J. Natl. Inst. Public Health.

[37] Yamamoto Y., Nomura Y., Okada A., Kakuta E., Yoshida N., Hosoya N., Hanada N., Takei N. (2021). Improvement of workplace environment that affects motivation of Japanese dental hygienists. Int. J. Environ. Res. Public Health.

[38] Nomura Y., Okada A., Miyoshi J., Mukaida M., Akasaka E., Saigo K., Daikoku H., Maekawa H., Sato T., Hanada N. (2018). Willingness to work and the working environment of Japanese dental hygienists. Int. J. Dent..

[39] Saito A., Tomita C., Sato Y., Cathcart G. (2009). Perceptions of Japanese and Canadian dental hygiene students towards their profession. Int. J. Dent. Hyg..

[40] World Economic Forum (2021). Global Gender Gap Report 2021.

[41] Patel B.M., Boyd L.D., Vineyard J., LaSpina L. (2021). Job satisfaction, burnout, and intention to leave among dental hygienists in clinical practice. J. Dent. Hyg..

[42] Araki J., Masaki I., Matsushima K., Date Y. (2017). A study on factors affecting career prospects for working women in Japanese enterprises. Jpn. J. Adm. Sci..

[43] Tano R., Miura H., Oshima K., Noritake K., Fukuda H. (2022). Relationship between career education experience among final-year dental hygiene students and their perspective towards work and profession: A nationwide survey in dental hygiene schools of Japan. Int. J. Dent. Hyg..

[44] Kamiura T., Ogasawara T., Masuda Y., Tomida M. (2020). Factors involved in intention to continue employment as a dental hygienist: Relationship with external career and internal career. Jpn. J. Dent. Prac. Admin..

